# Perceived nutrition needs of people experiencing disadvantage in utilizing support services: An Australian case study

**DOI:** 10.1002/fsn3.4072

**Published:** 2024-03-06

**Authors:** Verena T. Vaiciurgis, Annabel K. Clancy, Grace O'Reilly, Eleanor J. Beck

**Affiliations:** ^1^ School of Medical, Indigenous and Health Sciences University of Wollongong Wollongong New South Wales Australia; ^2^ Illawarra Health & Medical Research Institute Wollongong New South Wales Australia; ^3^ School of Health Sciences University of New South Wales Kensington New South Wales Australia

**Keywords:** diet‐related health inequalities, nutrition, socio‐economic disadvantage

## Abstract

Individuals experiencing socio‐economic disadvantage face higher rates of food insecurity and health disparities. This study explored the perceptions, attitudes, and knowledge of individuals providing nutrition services, and users of these services, to identify nutrition needs and inform potential strategies for addressing diet‐related health inequities. Semi‐structured interviews were conducted utilizing a phenomenological approach to explore lived experiences, beliefs, and perceptions influencing nutrition‐related health. Key themes were derived by consensus among researchers using inductive thematic analysis. Twenty‐two interviews were completed, which identified five themes. “Budgetary Constraints” was found to have a pervasive impact on all nutrition‐related services. Secondly, diverse “Individual Clientele” was found to influence three overlapping themes pertaining to opportunities and limitations for “Knowledge and Skills,” “Services, Resources and Staff,” and the “Systems and Food Environment.” Budgets directly impact the availability of services, resources, food provision, sustainability, and educational opportunities for staff, volunteers and service users. A live‐in environment offers a platform to implement and evaluate targeted interventions to inform and enhance nutrition‐related support services. Future interventions should address individual and systemic influences, prioritizing client‐informed, cost‐effective, sustainable capacity building for clients and staff. Recommendations for systemic and environmental influences include formalized staff training, peer‐mentoring systems, and increasing client autonomy. This has the potential to improve food security for residents following their transition into independent living. Charitable system limitations underscore the need for broader systemic change, informed policymaking, and government intervention to effectively address the root causes of food insecurity and diet‐related health inequities.

## Graphical abstract

Individuals experiencing socio‐economic disadvantage face higher rates of food insecurity and diet‐related health disparities. Addressing these issues requires identifying barriers and devising strategies that encompass individual and systemic factors and prioritizing client‐informed, cost‐effective, and sustainable models for improving nutrition access and health equity.

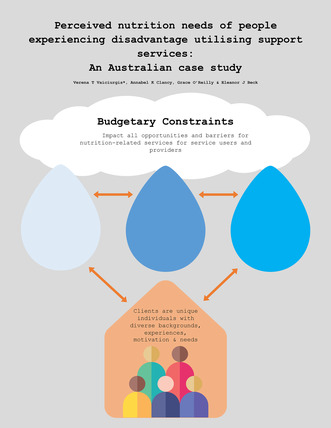

## 1 INTRODUCTION

Socio‐economic disadvantage is a key driver of food insecurity and health inequities, which are increasingly seen in Australia and globally (McKay et al., 2019; Pollard et al., 2018). These disparities are heightened in people living with disadvantage including but not limited to individuals experiencing or at risk of homelessness (Stafford & Wood, 2017). Compared to the general population, homeless individuals experience significantly higher prevalence of mental and physical illness, and alcohol and/or substance abuse, which are exacerbated by limited access to health services (Fazel et al., 2014). Furthermore, “marginalized” communities disproportionately experience poor nutritional intake and subsequently are more susceptible to poor diet‐related health outcomes such as malnutrition, metabolic comorbidity, and early mortality (Munialo & Mellor, 2023; Pettinger et al., 2017).

Food insecurity further affects the nutrition and health status of individuals unable to afford and/or access safe and nutritionally adequate food (McKay et al., 2019), extending to limitations in food literacy, storage, and preparation facilities (Albrecht, 2017; Sprake et al., 2014). Charitable food systems (CFS), including food banks and meal services, are the primary response to food insecurity in Australia (Booth et al., 2018; Lindberg et al., 2015; Pollard et al., 2018), providing valuable emergency food relief. However, studies indicate a lack of food literacy among both food‐insecure individuals (Ankrah et al., 2023; Begley, et al., 2019; Begley et al., 2019a, 2019b; West et al., 2020) and the staff and volunteers involved in charitable food provision (Lawlis et al., 2019). Recent research in Australia suggests that while staff and volunteers receive extensive training in emergency relief, training in nutrition and food security is limited (Albrecht, 2017; Lindberg et al., 2015), and the food provided by CFS may not meet the nutritional needs of clients (Roth, 2020; Simmet et al., 2017; Tse & Tarasuk, 2008). However, research into effective strategies to address the nutrition‐related drivers of health inequities in populations experiencing disadvantage is limited, and little has been done in collaboration with service providers and service recipients (Sprake et al., 2014; Wicks et al., 2006). Despite this gap, existing evidence highlights the importance of incorporating community perspectives in designing interventions for priority communities (Buck et al., 2004; Devine & Lawlis, 2019; Ijaz et al., 2018; Liamputtong, 2006).

To address these existing research gaps, this study aimed to actively collaborate with and gather perspectives from residents, drop‐in service users, staff, and volunteers at a center for homeless men in Sydney, Australia. The primary objective was to identify areas for improving nutrition‐related health outcomes for all clients.

Conducted within a broader co‐design framework, findings will be used to inform future research and develop strategies to address key drivers of nutrition‐related health inequities within a supported environment. The center, funded by both government and charitable donations, provides comprehensive residential and drop‐in services for homeless or at‐risk individuals. Notably, the structured weekday meal preparation system, integrated into its living skills program, offers residents a transiently more secure food environment. This study provides a unique opportunity to better understand the factors influencing nutritional health in this specific group, contributing valuable insights to the broader understanding of effective strategies for marginalized populations.

## 2 METHODS

This qualitative study conducted semi‐structured interviews in two phases: firstly, internal employees and health professional volunteers, then residents and drop‐in service users. The study was approved by a local institutional Human Research Ethics Committee. The reporting of findings adhered to the guidelines outlined in The Consolidated Criteria for Reporting Qualitative Research (COREQ) (Tong et al., 2007).

Participant eligibility criteria included individuals aged 18 or older who were either current or past (within the last 2 years) service providers or residents at the center. All staff and volunteers, including management, health professionals, individuals involved in food procurement, and case workers, received invitations to participate in the study via email. Initial recruitment of residents was guided by purposive sampling, facilitated by the Professional Services Coordinator at the center. Those utilizing residential services were requested to express their initial interest to the Professional Service Coordinator, via physical recruitment flyers. This provided a platform for addressing questions and concerns with a familiar and trusted person. People expressing interest in engaging in the study were subsequently contacted directly by the research team (VV and GO) to arrange an interview at a time and location convenient to the participant.

A phenomenological approach was utilized with predominately open‐ended questions which allowed for collection of detailed data and flexibility to establish experiences, beliefs, and perceptions regarding the underlying factors influencing nutrition‐related health and well‐being for individuals experiencing disadvantage, including potential strategies for improving support. This approach was chosen as the service providers and service users were considered experts in their lived experience. Questions were informed by a review of background literature and existing nutrition‐related services (Table 1). Probing questions were used to encourage participants to expand or clarify views expressed. Contemporaneous analysis of interviews allowed cessation of interviews when no new themes were identified.

**TABLE 1:** Semi‐structured interview question and probe guide.

Staff and volunteers	Residents
How often do you/did you work at X? What services do you/did you provide? Do you provide any food/beverages as part of your services? Are you aware of any nutrition‐related problems that clients may be experiencing? (*Probe, high cholesterol, poor dentition, problems with purchase of food, and cooking skills*) Do clients ever ask nutrition‐related questions? (*Follow up – what are some of the questions?*) Do you believe you have adequate knowledge or skills to assist in these problems? (*Follow up—What might you do to respond?*) Have you ever received any nutrition‐related education/training? (*Follow up—describe*) Are you aware of the dietetics service offered at X? (*If yes—probe do they refer to? Have they received any feedback?*) Are you aware of the food services provided at X? (*If yes—prompt have they received any feedback?*) Do you have any ideas on what nutrition‐related services could be helpful and offered as part of X services?	How long have you lived at X}?—Have you lived in similar places? Do you eat all of your meals here?—Where else would you eat? Are you satisfied with the food provided & prepared—Amount, quality, variety, and preparation? The System: Individuals take turns to cook—satisfied? Do you have all ingredients you need? Do you feel you have the necessary skills to cook? Who provides guidance on food here? Have you had any concerns/questions? Are you satisfied with your personal diet? Do you think it needs any changes to be healthier? Has a health professional ever told you to make dietary changes?—Doctor, dietitian, and social worker. Have you ever seen a dietitian? If you had a question about food/nutrition, where would you seek information?—Google, doctor, dietitian, and case worker? We are really interested in improving food here at X but also education/services for while you're here AND once you leave. Any suggestions—Prompt—Cooking classes, education sessions, shopping tours, recipe books, etc.

Interviews were conducted face‐to‐face or via videoconferencing platform (Zoom Video Communications Inc., 2020) and moderated by student researchers (removed for peer review) with guidance from experienced qualitative researchers who also hold dietetics qualifications (removed for peer review). All dialogue for interviews was audio‐recorded using two recorders, transcribed automatically by Zoom or Otter.AI software, and then checked/corrected for data authenticity, accuracy, and consistency. Transcribed data were coded and analyzed using an inductive approach of the six‐phase method described by Braun and Clarke (2006) utilizing software program NVivo 12 (QSR International Pty, Ltd, Melbourne, Australia, 2019). Transcribed data were read independently by two researchers for familiarity and to generate initial codes systematically across the datasets (VV all data, GO, AC, and EB duplicate). These codes were then collated into candidate themes, which were reviewed using the two‐level method to ensure accuracy and cohesion, and then further refined via discussions with the research team to reach a consensus on key themes.

## 3 RESULTS

A total of 22 interviews were completed (staff/volunteers *n* = 12, residents *n* = 10). Staff and volunteers (male *n* = 5, female *n* = 7), comprised of current employees (*n* = 6), current volunteers (*n* = 3), previous health professional volunteers (*n* = 2), and an individual funded by a local health district (*n* = 1). Caseworkers held social work qualifications, however, were not clinically trained (*n* = 6). All volunteers were clinically trained health professionals (*n* = 6) and four of these reported some education in nutrition. Two were dietitians and two others reported receiving limited training in nutrition in their health professional education. Ten interviews were also undertaken with male participants, (*n* = 10) residing at the center at the time. The length of time participants had been residents ranged from 2 weeks to 2 years. No drop‐in service users were recruited as drop‐in services were temporarily disbanded due to the COVID‐19 pandemic after the original planning of this research.

**FIGURE 1:**
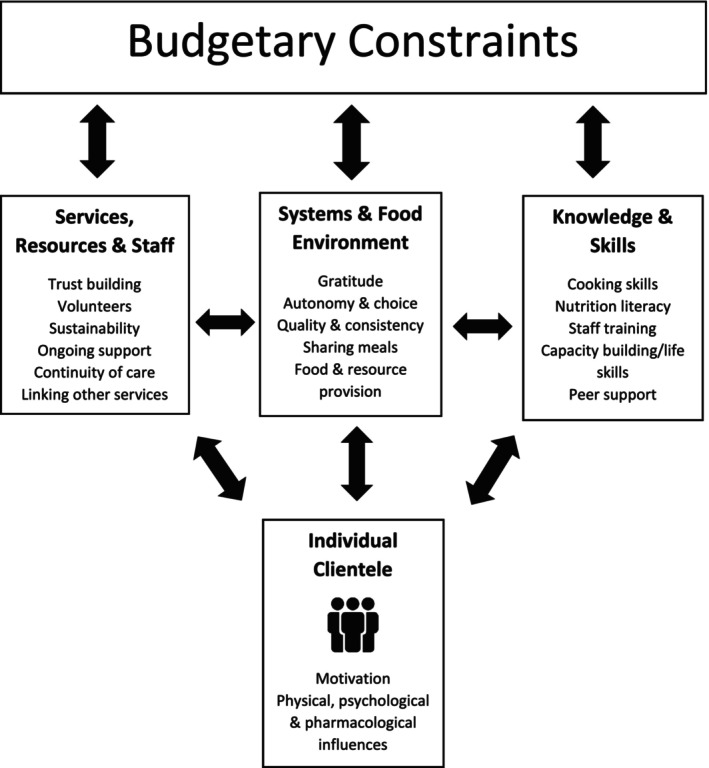
Concepts and related themes emerging from interviews with staff, volunteers, and residents of a center for homeless men.

Overall, participant interviews revealed five key themes (Figure 1) related to nutrition‐related services. An overarching theme of “Budgetary Constraints” was found to impact all nutrition‐related services and issues identified by participants, to some extent. The unique and diverse nature of the “Individual Clientele” was found to influence the remaining three themes pertaining to opportunities and limitations on “Knowledge and Skills,” “Services, Resources and Staff,” and the “Systems and Food Environment” at the center, with several overlaps identified.

All nutrition‐related services were found to be affected by *Budgetary Constraints*. This included staffing, client food provision, and education at the center, as well as the importance of long‐term budgeting for clients. For example, staff emphasized client food purchasing is driven by consistency and affordability for the center but also modeling sustainability for clients when they transition into independent living.

the plan is for them to be independent and self‐sufficient and, you know, that's why we don't have extravagant meats and, like, really high end food because … one it's like, too expensive and two when they leave they can't afford that stuff. (STAFFID 06)

All participants further noted that these constraints adversely affect clients' autonomy and choice, as well as the quality and variety of food provided to them.

… I saw them down at the supermarket on special half price and I thought well why don't we just get two or three boxes… I said maybe you can reimburse me but nah I can't do that, I'll have to take that to management and it's like just something simple like that, it's like you know I know everyone eats it on the level… Instead of getting stuff that we are not going to eat… so it's hard to sort of ask for something different. (RESIDENTID 06)

A perception of healthy eating as more expensive was expressed, in addition to budgetary constraints limiting the availability of resources and services that could help clients improve their nutrition status and literacy during and after residing at the center.

if you've only got, $40 a week for food, it's pretty hard to eat a decent diet on that limited amount. (STAFFID 09)

…if you're talking about a limited budget yeah I mean you talk about getting the two dollar fifty sausages and pasta and 60 cent pasta sauce, you know what I mean, you might not have the money for that, you might want to add veggies to that you know I would love to add some capsicum and some onion and you know what I mean but I'm on a four dollar budget for a meal tonight … (RESIDENTID 07)

Participants also noted that the center's health services are highly dependent on volunteers, and appointment availability is often insufficient. Clients have limited disposable income and are reliant on these free services to improve their health as well as their nutritional and financial literacy.

they will have a very limited access to getting educated about nutrition and it's been hard enough for the dietitians there to keep up with a residents. (STAFFID 02)

Well actually shopping, shopping is good one. I'd normally go and buy a big box of meat and then I'd go down to the shop and just buys set things. I wouldn't look around the shop and buy anything else… a lot of people that are on Centrelink (Australian welfare payment), do find it difficult to budget their meals and everything else. I usually spent, you know when I got back on to Centrelink. I was spending $15 a night. Just for dinner. Going to the supermarket, the prices have gone up as well in the way of meat and everything else. (RESIDENTID 09)

A second overarching theme focused on the diverse knowledge, skills, experiences, needs, and challenges of *Individual Clientele*. Staff identified clientele as an at‐risk group with multiple barriers to nutrition‐related health and well‐being, including homelessness, low socioeconomic status, metabolic comorbidities, specialized dietary requirements, substance use disorders, mental health disorders, and nutrition consequences of psychotropic medications. Clientele have highly varied levels of intrinsic motivation and access to affordable, appropriate support services, making nutrition a low priority. In addition, clients have significant disparities in skills, knowledge, and education, further contributing to differing levels of motivation. For example, clients ranged from qualified chefs to individuals with no cooking experience.

the thing is, so we look at everything holistically, we don't just address just one difficulty or one barrier that a client is experiencing as you guys know now, our clients have multiple barriers… We encourage them to work on everything at the same time, little bit at a time… we help them set and achieve their goals. (STAFFID 07)

I think most of them experience nutrition related issues, because of recently being homeless a large percentage have, quite severe drug and alcohol issues that are currently being addressed. Most of them, have had poor nutrition through lack of finances lack of kitchen lack of knowledge. (STAFFID 09)

… once again the main hurdle that they face is finding motivation for people to get the motivation to wanna learn… (RESIDENTID 01)

Participants also noted that a dislike of cooking or lack of motivation was often linked to confidence or a fear of cooking. To address this, clientele are usually paired with more experienced peers for support, which was reported to improve cooking confidence and skills.

we have guys that have never been or have come in and say, “I just don't know how to cook” and that's really common and it's a fear cooking rather than a dislike of cooking it's usually disguised as a dislike, but really it's just because they don't know how to cook. (STAFFID 01)

Well I'm a hopeless cook. I must admit my late wife, she was a chef so when she was alive… I wasn't allowed in the kitchen and all that, so coming here I was a little bit, oh woah… But there's someone always willing to show you… I'm looking forward to it now. Yeah. [NAME] on our floor. He was once a cook. Well still is, he will no worries be by your side. And it's wonderful. (RESIDENTID 10)

Although the diverse backgrounds of clients were found to be a significant consideration for the center, it is equally applicable to staff and volunteers. All participants unanimously agreed that *knowledge*, *skills*, and *education* are vital to empowering both clients and staff and improving the food system and environment. The center's independent living skills program incorporates essential skills such as cooking, shopping, and budgeting and is noted to be linked to improved self‐esteem and confidence in clients. However, the only formal nutrition services available are infrequent dietetics services and the occasional cooking class run by volunteers.

While staff recognized the importance of food preparation skills in improving the nutritional status of clients, they also reported having no formal nutrition education. Staff expressed learning by experience and provision of some form of dietary guidance to their clients. This was not necessarily when asked directly about nutrition or cooking, but tacitly in their guidance in cooking. There was an overwhelmingly positive opinion expressed toward learning more and imparting this knowledge to clients, although they had not had that opportunity.

It's something that I enjoy talking about, but I've never had any, any training… I mean, I would definitely be interested in it, and I think it'd be fantastic to have that information to share. (STAFFID 01)

Participants noted the existing group cooking setting as an ideal platform for integrating additional support. Many clients are familiar with and comfortable engaging in groups, making it a more efficient use of resources and time. In addition, the group setting promotes peer‐to‐peer encouragement and learning. Some participants suggested that a peer support program, which leveraged residents' diverse backgrounds and education levels, would be extremely valuable in this setting. This approach was also identified as an opportunity to build connections, and foster a sense of community and trust at the center. All participants provided specific ideas on how to improve nutrition knowledge and skills, with practical classes in a group setting being a common suggestion. Other suggestions included cooking and shopping for good health on a budget and the provision of simple written resources.

Yeah, I found they're (cooking classes) quite good … there was a good group of us that used to go … it's more motivating and also provides a connection with people here. (RESIDENTID 01)

a nutrition group, whether that's run by a dietitian or whether it's, you know, facilitated by a student dietitians I guess ideally, if you had an employed dietitian even if it was, like, one day a week that they could be supervising groups or running those groups would be beneficial. (STAFFID 05)

written information, a lot of people don't have great Internet access or smart phones and stuff like that. So really straight forward information about nutrition as well as a few simple recipes and stuff like that. (STAFFID 09)

I'd like to learn a bit more with the ingredients that I can use to put in and make that actual sauce myself. Instead of just, actually, just Okay, I'm going to pull chicken satay out of the cupboard. And done. That's why it's quick and fast for me, I'd rather start learning. (RESIDENTID 09)

Ensuring the *sustainability of staff*, *services*, and *resources* at the center was closely aligned with budget. The center faces several challenges in providing ongoing professional care. The health services are primarily provided by volunteers, which limits opportunities for capacity building and continuity of care due to sustainability of volunteers due to other commitments, time, and financial constraints. Given the clientele, ongoing care is of particular concern around managing mental health and sobriety. Participants suggested linking in with other services (such as government health services) may be useful to address this; however, this was often difficult due to the uncertainty of where the clients would be physically living once they leave.

it'd be nice if there was somebody that also could maybe come in and offer to teach us to cook or give us ideas on different meals, or that we can prepare with food that we're actually given. (RESIDENTID 09)

for as long as I've been there we've had volunteers. It's just that sometimes you know days change, commitments change with the volunteers and we've probably had about four or five since I've been there, different volunteers that will stay on for a few months or up to year even and just go through. (STAFFID 01)

So we used to run cooking classes, but it's been difficult to get proper people to come and do that all our clients used to enjoy that sort of group activity and also someone, you know, take them shopping. (STAFFID 07)

The final theme related to the unique *Systems and Food Environment*. The center's holistic system aims to develop residents' independence and life skills, empowering them once they have moved into the community. Responses to this system varied greatly among interviewees. Some participants expressed gratitude for the extensive programs, cooking and cleaning systems, sense of community, and supportive staff. Conversely, some participants felt they were left to their own devices and lacked support, describing a lack of community and expressing that the rules were not enforced. Staff interviewees acknowledged that food systems are interrelated to sustainable living skills and the center's budget. They also noted that while this arrangement may not suit everyone, it does allow individuals to utilize their existing skills and successfully prepare clients with additional independent living skills, such as long‐term budgeting.

Residents and caseworkers, in particular, noted that clients influence and encourage each other by providing a sense of family or community. There were also some small opportunities for food used in celebration which added a sense of community. Although food is considered primarily around its teaching as a core life skill, center staff still recognize the social and cultural significance of food in celebration.

## 4 DISCUSSION

The current study found financial constraints severely limit the availability and sustainability of services, resources, staff, and opportunities to improve the knowledge and skills of both clients and staff, as well as the food system and environment at the center. This finding aligns with previous studies in Australia (Bortolin et al., 2018) and the United States (Koh et al., 2016) that found budgetary constraints to be the most significant barrier to accessing and providing nutritious foods.

The client group was identified as “at‐risk” with multiple barriers to adequate nutrition, including diverse nutrition‐related knowledge, lack of finances, and living skills. Participants perceived that nutrition education for both staff and clients would be beneficial for improving client health outcomes. However, participants also believed budgetary constraints are restricting such opportunities, largely due to the limited availability of services and resources, vastly dependent on already inadequate numbers of time‐poor volunteers. Although provision of meals at a facility was found to remove some of the barriers around access to food, storage, and preparation facilities, the results here are also consistent with previous research findings that food provision is managed by staff who have had no formal training in nutrition or food insecurity (Devine & Lawlis, 2019; Scouten et al., 2016). Despite this lack of training and commitment of staff and volunteers, their ability to effectively support clients may be limited by their lack of necessary knowledge and skills. While nutrition knowledge and skills can motivate clients to make changes, additional barriers can hinder clients in their ability to sustain their learned skills and prioritize their nutrition‐related health.

Where disparate views existed, participants still articulated similar solutions for these problems. As identified in previous research (Levi et al., 2022), participants acknowledged there are existing intrinsic and extrinsic factors that influence individuals' food access preparation, planning, and dietary intake (Levi et al., 2022). These factors are inextricably linked and thus, interventions must consider client motivation and readiness to change. Furthermore, cost‐effective strategies, including group interventions, could be employed to enhance the capacity of both staff and clients, thereby improving overall food systems and the environment within the center.

Notably, a growing body of evidence highlights the inherent limitations of food relief systems, as despite well‐intentioned efforts, they are unable to address the broader drivers of food insecurity and diet‐related health inequities (Zack et al., 2021), including the insufficient availability of reliable financial, social, and food resources (Pollard et al., 2018). Consequently, there is increasing recognition of the need for extensive, system‐wide political intervention, support, and collaboration to effectively address these complex issues (Inza‐Bartolomé, 2022; Schwartz & Caspi, 2023). Overall, factors influencing nutrition‐related health and support are complex, with varying perspectives highlighting the need for ongoing intervention, evaluation, and improvement. Charitable systems, however, are integral to navigating this complex landscape and could benefit from government assistance, collaboration, and support (Zack et al., 2021).

Another key barrier identified to adequate nutrition was a lack of finances and budgeting skills in addition to a perception that healthy eating is more expensive and subsequently unattainable. An Australian study using a standardized affordability and pricing method (ASAP) found that a healthy diet is actually 15%–17% less expensive, however, often unaffordable for low‐income households as the costs are equal to 30% of an average disposable income (Lee et al., 2018). The Healthy Food Access Basket (HFAB) survey, assessing the affordability of a healthy diet in line with the Australian Guide to Healthy Eating, reported a lower average of 20.6% for a one‐person household on government assistance only, and 16.5% for the cheapest (generic) HFAB with the largest contributor coming from lean meats, eggs, nuts, and seeds (Queensland Health and Queensland Treasury, 2023). At the center, cheaper cuts of meat are provided daily, despite animal‐based proteins being more expensive than nonanimal protein sources. A reduction in animal protein in some/or all of the meals has the potential to reduce this proportion further (Flynn & Schiff, 2015). Other studies suggest that improvements in dietary quality are possible within a constrained budget, by substituting refined grains with whole grains and low saturated/trans fat substitutes (Koh et al., 2016).

The limited availability of services, resources, staff, and training opportunities for clients and staff also posed a significant barrier to promoting nutrition‐related health. However, group interventions for clients and staff involved in food provision have the potential to improve the nutrition status of clients. This approach can reach larger numbers of people and thus may be more cost‐effective and time‐efficient use of resources and volunteers' services. For example, individualized dietitian services could be redirected to food literacy training with staff and clients. Group education has also been shown to promote peer support, peer‐to‐peer learning, and social skills and could include components supporting additional modifiable health behaviors (Aschbrenner et al., 2016). Nutrition education programs aimed at improving food literacy, especially interventions with a cooking component, are linked with improved skills, confidence, knowledge, dietary intake, and food security, particularly in low‐socioeconomic adult populations (Begley et al., 2019; Begley et al. 2019a, 2019b; Reicks et al., 2018; West et al., 2020). Food literacy interventions focus on planning, selection, and preparation to promote eating behaviors, typically including budgeting tips and preparing budget‐friendly meals (Begley et al., 2019; Begley et al. 2019a, 2019b). Communal cooking provides an opportunity for this type of program and has the added advantage of potentially combatting the perception that healthy eating is more expensive (Lee et al., 2020).

Many participants also noted that residents at the center who were more knowledgeable would usually provide advice and assistance to those who lacked this food and nutrition education. Most agreed that they enjoyed this, as it provided connection and empowerment, contributing to the sustainability of knowledge over time. However, this system seemed to be casual and unstructured. A more formalized peer–mentor structure could be a cost‐effective model easily integrated into the existing system. Several studies have explored the advantages of using a peer–mentor system to deliver education and successfully change health behaviors, particularly in vulnerable communities (Anderson et al., 2005; Barker et al., 2018; Erwin et al., 2017; Oliver et al., 2020; Petosa & Smith, 2014). Both mentors and mentees experienced satisfaction, a sense of connection, empowerment, and improved health outcomes.

Another major influence supporting nutrition‐related health was the diversity in client motivation and needs. This included mental health conditions and associated psychotropic medication side effects, existing nutrition beliefs, and substance and tobacco use. Addiction and mental health conditions are prominent in homeless communities, which have direct influence on nutrition status (Booth et al., 2018; Brown et al., 2019; Johnson et al., 2009; Lindberg et al., 2019). These influences must be considered in any potential intervention, for example, low preparation meals and smoking cessation. An opportunity, as part of a holistic framework, exists in residential settings to provide programs targeted at improving modifiable health behaviors to prevent and manage chronic disease, mental health, and addiction. Motivational interviewing and referring to The Transtheoretical Model “Stages of Change” could be beneficial in this setting (Miller & Rollnick, 2012). Integrating health and nutrition education into CFS has proven successful globally (Cheyne et al., 2020; Lawlis et al., 2019; Meiklejohn et al., 2017). Particularly, when involving health professionals such as dietitians, and community partnerships with higher education sectors (Devine & Lawlis, 2019; Lawlis et al., 2019).

The rules and systems governing organizations can have a profound impact on food and nutrition environments. In the present study, staff are responsible for all food ordering, and residents operate on a weeknight cooking schedule. Many residents expressed difficulty in developing life skills due to limited autonomy and choice. Therefore, future interventions should consider giving residents more responsibility and autonomy in food purchasing decisions.

Other participant ideas to improve nutrition‐related services included the provision of written resources encompassing nutrition information, for example, recipes, basic shopping lists/tours, measurements, cooking techniques, references to information/services, label reading, and budgeting. These ideas could be delivered via a food literacy program and could integrate other participant ideas unrelated to food provision such as improved communication between current health professionals; linking in with other services; coordination of care; and specific nutrition training for mental health.

This study, while identifying and exploring potential opportunities for intervention, is inherently limited by its phenomenological design which is not suitable to all health research questions. The findings are limited to the subjective lived experiences, biases, and perspectives of service providers, male‐only residents (within one center), and researchers. The relatively small sample size used for interviews, along with the motivated nature of participants, introduces potential biases including participant, social desirability, and content bias. Thus, caution must be exercised in generalizing the findings beyond this specific context, as the subjective nature of phenomenology, inherent biases, and the lack of control in experimental designs may compromise the broader applicability of the results. Future research could utilize additional research methodologies (e.g., descriptive qualitative design, with highly structured questions aimed at eliciting participants' views) to address limitations and advance the quality and applicability of research in this area.

## 5 CONCLUSIONS

Given the high, inequitable prevalence of mental health and metabolic disease, access to adequate nutrition and appropriate health services must be considered a priority for individuals experiencing disadvantage. The individual nature of the “client” must be recognized to maintain respect, and meet individual needs. Both service providers and users face multiple barriers in providing and receiving support. Overall, this study indicates budget has direct impact on systems and food environment including availability of staff, resources, services, food provision, and opportunities for client, staff, and volunteer training. A live‐in environment provides a unique setting to implement and evaluate targeted interventions to inform and enhance nutrition‐related services. Future research should focus on client‐informed, cost‐effective, sustainable models for nutrition‐specific capacity building for both individual clients and staff. Strategies to improve existing systemic and environmental influences could involve formalized staff training, peer‐mentoring systems, and increasing autonomy and choice for clients. The limitations of charitable systems highlight the imperative for broader systemic change, informed policymaking, and active government intervention to effectively address the underlying drivers of food insecurity and diet‐related health inequities.

## AUTHOR CONTRIBUTIONS

**Verena T. Vaiciurgis:** Conceptualization (equal); data curation (equal); formal analysis (equal); investigation (equal); methodology (equal); writing – original draft (lead); writing – review and editing (equal). **Annabel K. Clancy:** Conceptualization (lead); formal analysis (equal); methodology (equal); project administration (equal); supervision (equal); writing – review and editing (equal). **Eleanor J. Beck:** Conceptualization (equal); formal analysis (equal); methodology (equal); project administration (equal); supervision (equal); writing – review and editing (lead). **Grace O'Reilly:** Data curation (equal); formal analysis (supporting); investigation (equal); methodology (equal); writing – original draft (supporting); writing – review and editing (supporting).

## CONFLICT OF INTEREST STATEMENT

The authors declare no conflicts of interest.

## ETHICS STATEMENT

This study was conducted according to the guidelines laid down in the Declaration of Helsinki and all procedures involving research study participants were approved by the University of Wollongong Human Research Ethics Committee (Approval number 2019/429). Written informed consent was obtained from all subjects/patients.

## Supporting information


Appendix S1

## Data Availability

On reasonable request, the corresponding author will provide the datasets created and used in the current study.

## References

[fsn34072-bib-0001] Albrecht, K. A. (2017). *Nutritious meals for the homeless population: Challenges and opportunities* (Masters theses). 3200. Retrieved from https://thekeep.eiu.edu/theses/3200

[fsn34072-bib-0002] Anderson, A. K. , Damio, G. , Young, S. , Chapman, D. J. , & Pérez‐Escamilla, R. (2005). A randomized trial assessing the efficacy of peer counseling on exclusive breastfeeding in a predominantly Latina low‐income community. Archives of Pediatrics & Adolescent Medicine, 159(9), 836–841. 10.1001/archpedi.159.9.836 16143742

[fsn34072-bib-0003] Ankrah Twumasi, M. , Essilfie, G. , Ntiamoah, E. B. , Xu, H. , & Jiang, Y. (2023). Assessing financial literacy and food and nutritional security relationship in an African country. Heliyon, 9(9), e19573. 10.1016/j.heliyon.2023.e19573 37809661 PMC10558823

[fsn34072-bib-0004] Aschbrenner, K. A. , Naslund, J. A. , & Bartels, S. J. (2016). A mixed methods study of peer‐to‐peer support in a group‐based lifestyle intervention for adults with serious mental illness. Psychiatric Rehabilitation Journal, 39(4), 328–334. 27560454 10.1037/prj0000219 PMC5125856

[fsn34072-bib-0005] Barker, S. L. , Maguire, N. , Bishop, F. L. , & Stopa, L. (2018). Peer support critical elements and experiences in supporting the homeless: A qualitative study. Journal of Community & Applied Social Psychology, 28(4), 213–229. 10.1002/casp.2353

[fsn34072-bib-0006] Begley, A. , Paynter, E. , Butcher, L. M. , Bobongie, V. , & Dhaliwal, S. S. (2019). Identifying participants who would benefit the most from an adult food‐literacy program. International Journal of Environmental Research and Public Health, 16(7), 1272. 30970671 10.3390/ijerph16071272 PMC6480264

[fsn34072-bib-0007] Begley, A. , Paynter, E. , Butcher, L. M. , & Dhaliwal, S. S. (2019a). Effectiveness of an adult food literacy program. Nutrients, 11(4), 797. 30959958 10.3390/nu11040797 PMC6520903

[fsn34072-bib-0008] Begley, A. , Paynter, E. , Butcher, L. M. , & Dhaliwal, S. S. (2019b). Examining the association between food literacy and food insecurity. Nutrients, 11(2), 445. 10.3390/nu11020445 30791670 PMC6412525

[fsn34072-bib-0009] Booth, S. , Begley, A. , Mackintosh, B. , Kerr, D. A. , Jancey, J. , Caraher, M. , Whelan, J. , & Pollard, C. M. (2018). Gratitude, resignation and the desire for dignity: Lived experience of food charity recipients and their recommendations for improvement, Perth, Western Australia. Public Health Nutrition, 21(15), 2831–2841. 10.1017/S1368980018001428 29947318 PMC6141991

[fsn34072-bib-0010] Bortolin, N. , Priestly, J. , & Sangster, J. (2018). Food provision among food relief agencies in rural Australia, and perceived barriers and enablers to provide healthy food. Australian Journal of Rural Health, 26(2), 86–92. 29131446 10.1111/ajr.12398

[fsn34072-bib-0011] Braun, V. , & Clarke, V. (2006). Using thematic analysis in psychology. Qualitative Research in Psychology, 3(2), 77–101. 10.1191/1478088706qp063oa

[fsn34072-bib-0012] Brown, M. A. , Gellatley, W. , Hoffman, A. , Dowdell, L. , Camac, A. , Francois, R. , Boston, B. , & Zekry, A. (2019). Medical complications of homelessness: A neglected side of men's health. Internal Medicine Journal, 49(4), 455–460. 30324639 10.1111/imj.14139

[fsn34072-bib-0013] Buck, D. S. , Rochon, D. , Davidson, H. , & McCurdy, S. (2004). Involving homeless persons in the leadership of a health care organization. Qualitative Health Research, 14(4), 513–525. 10.1177/1049732303262642 15068577

[fsn34072-bib-0014] Cheyne, K. , Smith, M. , Felter, E. M. , Orozco, M. , Steiner, E. A. , Park, Y. , & Gary‐Webb, T. L. (2020). Food bank‐based diabetes prevention intervention to address food security, dietary intake, and physical activity in a food‐insecure cohort at high risk for diabetes. Preventing Chronic Disease, 17, E04. 10.5888/pcd17.190210 31922370 PMC6977780

[fsn34072-bib-0015] Devine, A. , & Lawlis, T. (2019). Nutrition and vulnerable groups. Nutrients, 11(5), 1066. 10.3390/nu11051066 31091644 PMC6566763

[fsn34072-bib-0016] Erwin, C. M. , McEvoy, C. T. , Moore, S. E. , Prior, L. , Lawton, J. A. , Patterson, C. C. , Kee, F. , Cupples, M. , Young, I. S. , Appleton, K. , McKinley, M. C. , & Woodside, J. V. (2017). Process evaluation of a complex intervention: Trial to encourage adoption and maintenance of a MEditerranean diet (TEAM‐MED). The Proceedings of the Nutrition Society, 76(OCE3), E112. 10.1017/S0029665117001859

[fsn34072-bib-0017] Fazel, S. , Geddes, J. R. , & Kushel, M. (2014). The health of homeless people in high‐income countries: Descriptive epidemiology, health consequences, and clinical and policy recommendations. The Lancet, 384(9953), 1529–1540. 10.1016/S0140-6736(14)61132-6 PMC4520328 25390578

[fsn34072-bib-0018] Flynn, M. , & Schiff, A. (2015). Economical healthy diets (2012): Including lean animal protein costs more than using extra virgin olive oil. Journal of Hunger & Environmental Nutrition, 10, 1–16. 10.1080/19320248.2015.1045675

[fsn34072-bib-0019] Ijaz, S. , Thorley, H. , Porter, K. , Fleming, C. , Jones, T. , Kesten, J. , Mamluk, L. , Richards, A. , Marques, E. M. R. , & Savović, J. (2018). Interventions for preventing or treating malnutrition in homeless problem‐drinkers: A systematic review. International Journal for Equity in Health, 17(1), 8. 10.1186/s12939-018-0722-3 29338739 PMC5771104

[fsn34072-bib-0020] Inza‐Bartolomé, A. (2022). The clash between charitable food and the human right to food. In L. Escajedo San‐Epifanio & E. M. Rebato Ochoa (Eds.), Ethics of charitable food: Dilemmas for policy and practice (pp. 137–149). Springer.

[fsn34072-bib-0021] Johnson, L. J. , Myung, E. , McCool, A. C. , & Champaner, E. I. (2009). Nutrition education for homeless women‐challenges and opportunities: A pilot study. Journal of Foodservice Business Research, 12(2), 155–169. 10.1080/15378020902910496

[fsn34072-bib-0022] Koh, K. A. , Bharel, M. , & Henderson, D. C. (2016). Nutrition for homeless populations: Shelters and soup kitchens as opportunities for intervention. Public Health Nutrition, 19(7), 1312–1314. 10.1017/s1368980015002682 26434381 PMC10271043

[fsn34072-bib-0023] Lawlis, T. , Sambell, R. , Douglas‐Watson, A. , Belton, S. , & Devine, A. (2019). The food literacy action logic model: A tertiary education sector innovative strategy to support the charitable food sectors need for food literacy training. Nutrients, 11(4), 837. 10.3390/nu11040837 31013852 PMC6520867

[fsn34072-bib-0024] Lee, A. J. , Kane, S. , Herron, L.‐M. , Matsuyama, M. , & Lewis, M. (2020). A tale of two cities: The cost, price‐differential and affordability of current and healthy diets in Sydney and Canberra, Australia. International Journal of Behavioral Nutrition and Physical Activity, 17(1), 1–13. 10.1186/s12966-020-00981-0 32571334 PMC7309977

[fsn34072-bib-0025] Lee, A. J. , Kane, S. , Lewis, M. , Good, E. , Pollard, C. M. , Landrigan, T. J. , & Dick, M. (2018). Healthy diets ASAP – Australian standardised affordability and pricing methods protocol. Nutrition Journal, 17(1), 88. 10.1186/s12937-018-0396-0 30261887 PMC6161417

[fsn34072-bib-0026] Levi, R. , Schwartz, M. , Campbell, E. , Martin, K. , & Seligman, H. (2022). Nutrition standards for the charitable food system: Challenges and opportunities. BMC Public Health, 22(1), 1–13. 35287656 10.1186/s12889-022-12906-6 PMC8919136

[fsn34072-bib-0027] Liamputtong, P. (2006). Researching the vulnerable: A guide to sensitive research methods (pp. 1–256). Sage.

[fsn34072-bib-0028] Lindberg, R. , McCartan, J. , Stone, A. , Gale, A. , Mika, A. , Nguyen, M. , & Kleve, S. (2019). The impact of social enterprise on food insecurity – An Australian case study. Health & Social Care in the Community, 27(4), e355–e366. 10.1111/hsc.12737 30848546

[fsn34072-bib-0029] Lindberg, R. , Whelan, J. , Lawrence, M. , Gold, L. , & Friel, S. (2015). Still serving hot soup? Two hundred years of a charitable food sector in Australia: A narrative review. Australian and New Zealand Journal of Public Health, 39(4), 358–365. 25716286 10.1111/1753-6405.12311

[fsn34072-bib-0030] McKay, F. H. , Haines, B. C. , & Dunn, M. (2019). Measuring and understanding food insecurity in Australia: A systematic review. International Journal of Environmental Research and Public Health, 16(3), 476. 10.3390/ijerph16030476 30736305 PMC6388276

[fsn34072-bib-0031] Meiklejohn, S. J. , Barbour, L. , & Palermo, C. E. (2017). An impact evaluation of the FoodMate programme: Perspectives of homeless young people and staff. Health Education Journal, 76(7), 829–841. 10.1177/0017896917715780

[fsn34072-bib-0032] Miller, W. R. , & Rollnick, S. (2012). Motivational interviewing: Helping people change. Guilford Press.

[fsn34072-bib-0033] Munialo, C. D. , & Mellor, D. D. (2023). A review of the impact of social disruptions on food security and food choice. Food Science & Nutrition, 12, 13–23. 38268897 10.1002/fsn3.3752 PMC10804122

[fsn34072-bib-0035] Oliver, T. L. , McKeever, A. , Shenkman, R. , & Diewald, L. K. (2020). Successes and challenges of using a peer Mentor model for nutrition education within a food pantry: A qualitative study. BMC Nutrition, 6(1), 1–27. 10.1186/s40795-020-00352-9 32685182 PMC7359598

[fsn34072-bib-0036] Petosa, R. L. , & Smith, L. H. (2014). Peer mentoring for health behavior change: A systematic review. American Journal of Health Education, 45(6), 351–357. 10.1080/19325037.2014.945670

[fsn34072-bib-0037] Pettinger, C. , Parsons, J. M. , Cunningham, M. , Withers, L. , D'Aprano, G. , Letherby, G. , Sutton, C. , Whiteford, A. , & Ayres, R. (2017). Engaging homeless individuals in discussion about their food experiences to optimise wellbeing: A pilot study. Health Education Journal, 76(5), 557–568.

[fsn34072-bib-0038] Pollard, C. M. , Mackintosh, B. , Campbell, C. , Kerr, D. , Begley, A. , Jancey, J. , Caraher, M. , Berg, J. , & Booth, S. (2018). Charitable food systems' capacity to address food insecurity: An Australian capital city audit. International Journal of Environmental Research and Public Health, 15(6), 1249. 29895801 10.3390/ijerph15061249 PMC6025598

[fsn34072-bib-0039] Queensland Health and Queensland Treasury . (2023). Affordability of healthy foods by household type in 2014 . Healthy Food Access Basket. Retrieved from https://www.health.qld.gov.au/research‐reports/reports/public‐health/food‐nutrition/access/affordability#one

[fsn34072-bib-0040] Reicks, M. , Kocher, M. , & Reeder, J. (2018). Impact of cooking and home food preparation interventions among adults: A systematic review (2011‐2016). Journal of Nutrition Education and Behavior, 50(2), 148–172.e1. 10.1016/j.jneb.2017.08.004 28958671

[fsn34072-bib-0041] Roth, S. E. (2020). Do nutrition policies matter? Assessing the determinants of nutritional quality of inventory at food banks. ProQuest Dissertations Publishing.

[fsn34072-bib-0042] Schwartz, M. B. , & Caspi, C. E. (2023). The charitable food system as a change agent. Frontiers in Public Health, 11, 1156501. 37064662 10.3389/fpubh.2023.1156501 PMC10102588

[fsn34072-bib-0043] Scouten, S. , Lucia, V. C. , Wunderlich, T. , Uhley, V. , & Afonso, N. M. (2016). An assessment of needs of church coordinators providing meals to a homeless shelter. Journal of Health Care for the Poor and Underserved, 27(3), 1211–1219. 27524763 10.1353/hpu.2016.0124

[fsn34072-bib-0044] Simmet, A. , Depa, J. , Tinnemann, P. , & Stroebele‐Benschop, N. (2017). The nutritional quality of food provided from food pantries: A systematic review of existing literature. Journal of the Academy of Nutrition and Dietetics, 117(4), 577–588. 27727101 10.1016/j.jand.2016.08.015

[fsn34072-bib-0045] Sprake, E. F. , Russell, J. M. , & Barker, M. E. (2014). Food choice and nutrient intake amongst homeless people. Journal of Human Nutrition and Dietetics, 27(3), 242–250. 10.1111/jhn.12130 23679134

[fsn34072-bib-0046] Stafford, A. , & Wood, L. (2017). Tackling health disparities for people who are homeless? Start with social determinants. International Journal of Environmental Research and Public Health, 14(12), 1535. 10.3390/ijerph14121535 29292758 PMC5750953

[fsn34072-bib-0047] Tong, A. , Sainsbury, P. , & Craig, J. (2007). Consolidated criteria for reporting qualitative research (COREQ): A 32‐item checklist for interviews and focus groups. International Journal for Quality in Health Care, 19(6), 349–357. 10.1093/intqhc/mzm042 17872937

[fsn34072-bib-0048] Tse, C. , & Tarasuk, V. (2008). Nutritional assessment of charitable meal programmes serving homeless people in Toronto. Public Health Nutrition, 11(12), 1296–1305. 10.1017/S1368980008002577 18547445

[fsn34072-bib-0049] West, E. G. , Lindberg, R. , Ball, K. , & McNaughton, S. A. (2020). The role of a food literacy intervention in promoting food security and food literacy‐OzHarvest's NEST program. Nutrients, 12(8), 2197. 10.3390/nu12082197 32718054 PMC7468773

[fsn34072-bib-0050] Wicks, R. , Trevena, L. J. , & Quine, S. (2006). Experiences of food insecurity among urban soup kitchen consumers: Insights for improving nutrition and well‐being. Journal of the American Dietetic Association, 106(6), 921–924. 16720134 10.1016/j.jada.2006.03.006

[fsn34072-bib-0051] Zack, R. M. , Weil, R. , Babbin, M. , Lynn, C. D. , Velez, D. S. , Travis, L. , Taitelbaum, D. J. , & Fiechtner, L. (2021). An overburdened charitable food system: Making the case for increased government support during the COVID‐19 crisis. American Journal of Public Health, 111(5), 804–807. 10.2105/AJPH.2021.306222 33826384 PMC8034014

